# Clinical predictors of early reflux and symptom severity after esophagectomy: a risk prediction tool

**DOI:** 10.1007/s10388-026-01225-z

**Published:** 2026-06-30

**Authors:** Zhao Xue, Liu Beijia, Xie Qin, Liu Jiarui, Wang Xiaowei, Luo Lei, Miao Yan

**Affiliations:** 1 Department of Thoracic Surgery, Sichuan Clinical Research Center for Cancer, School of Medicine, Sichuan Cancer Hospital & Institute, Sichuan Cancer Center, University of Electronic Science and Technology of China, Chengdu, Sichuan 610041 People’s Republic of China; 2 Department of Thoracic Surgery, Sichuan Clinical Research Center for Cancer, Sichuan Cancer Hospital & Institute, Sichuan Cancer Center, University of Electronic Science and Technology of China, Chengdu, Sichuan 610041 People’s Republic of China; 3 School of Nursing, Chengdu Medical College, Chengdu, Sichuan 610500 People’s Republic of China; 4 Department of General Office, Sichuan Clinical Research Center for Cancer, Sichuan Cancer Hospital & Institute, Sichuan Cancer Center, University of Electronic Science and Technology of China, Chengdu, Sichuan 610041 People’s Republic of China

**Keywords:** Esophageal Neoplasms, Gastroesophageal Reflux, Risk Prediction Models, Symptom Burden

## Abstract

**Background:**

Reflux after curative esophagectomy is common and may persist, disrupting eating, sleep, and recovery, yet most prediction tools estimate risk only at a single late time point.

**Methods:**

We retrospectively analyzed an esophagectomy derivation cohort (2018–2021; n = 488) and a temporal validation cohort (2023–2024; n = 143). Clinically significant reflux was defined as an EORTC QLQ-OES18 item 14 or 15 score ≥ 2. Time to first event within 12 months was modeled using a time-varying coefficient Cox model, and longitudinal symptom severity (1–4) was modeled using an ordinal mixed-effects model. Predictors were prespecified from routine pre-discharge variables. Model performance was assessed using time-dependent AUC and calibration.

**Results:**

Cox analysis linked distal/esophagogastric junction tumors and higher BMI to increased early reflux risk, while neoadjuvant therapy, older age, and a longer oral-to-solid diet interval were protective. The ordinal mixed model showed greater symptom severity in women and in patients with distal/esophagogastric junction tumors, but lower severity with neoadjuvant therapy. Interactions suggested slower early symptom escalation with older age and steeper escalation with higher BMI or shorter oral-to-intake intervals. The time-varying Cox model showed good discrimination, with time-dependent AUCs at 1, 3, and 6 months of 0.702, 0.766, and 0.794 in the development cohort, and 0.708, 0.767, and 0.716 in the external validation cohort, with good calibration.

**Conclusions:**

A pre-discharge, time-sensitive risk model with a web-based calculator may support risk-stratified early screening, education, and targeted supportive care after discharge to reduce reflux-related morbidity and protect postoperative health-related quality of life.

**Supplementary Information:**

The online version contains supplementary material available at https://doi.org/10.1007/s10388-026-01225-z.

## Background

Esophageal cancer remains a major global health challenge, with an estimated 511,054 new cases and 445,391 deaths worldwide in 2022 [1]. Although age-standardized mortality has declined since 1990, the absolute burden and survivorship needs remain substantial [2]. For resectable disease, esophagectomy remains a cornerstone of cure [3], but some surgery-related complications are unavoidable, with international benchmark data reporting an overall complication rate of about 59% [4]. As a serious, life-threatening malignancy, esophageal cancer leaves many survivors with persistent symptom burden after curative treatment. Reflux after esophagectomy is common and may persist, patient-reported reflux symptoms are frequent, but prevalence varies widely across cohorts and measurement approaches [5–7].

It reflects loss of anti-reflux barriers after removal of the lower esophageal sphincter and the angle of His, along with conduit denervation and altered thoracoabdominal pressure gradients, and it may be amplified by delayed conduit emptying [8]. Clinically, reflux often presents as a symptom cluster, including retrosternal burning, regurgitation, cough, and aspiration-related symptoms, and nocturnal or supine episodes can disrupt sleep and limit oral intake. In a nationwide cohort, severe reflux was associated with more sleep disturbance and worse health-related quality of life after esophageal cancer surgery. [9]. Long-term survey data suggest that reflux frequency decreases during the first two postoperative years and then plateaus, with a sizeable subgroup experiencing at least weekly symptoms and reporting worse global quality of life [10]. Yet follow-up after esophagectomy is usually standardized and often depends on history and physical examination, rather than risk-adapted, time-sensitive symptom surveillance [11]. Current prediction models have mostly targeted severe reflux at a single late time point [12].

Therefore, the primary objective of this study was to identify clinical predictors for both the timing of reflux onset and the longitudinal trajectory of its severity. Building on these findings, we aimed to develop and validate a time-sensitive risk prediction tool, presented as a user-friendly web-based calculator, to facilitate early identification of high-risk patients.

## Methods

### Study population and data collection

Adult patients who underwent curative esophagectomy at our institution between March 2018 and May 2021 were retrospectively enrolled to form the derivation cohort; a 2023–2024 cohort served for temporal validation. Eligibility criteria were age ≥ 18 years, histologically confirmed esophageal cancer, curative resection, and ≥ 1 postoperative EORTC QLQ-OES18 assessment including items 14 and 15; patients with 30-day mortality or reoperation were excluded. QLQ-OES18 was scheduled at 1, 3, 6, and 12 months. The study was ethics approved (SCCHEC-02–2020-004) with a waiver of informed consent. Candidate predictors were prespecified from the literature and clinical experience, and included demographics, baseline health and nutritional status, oncologic characteristics, surgical technique, and indicators of postoperative recovery.

### Surgical procedures and reflux outcomes

Esophagectomy and reconstruction followed institutional standards. Surgical data included the type of esophagectomy such as the three-incision McKeown procedure with cervical anastomosis or the Ivor Lewis approach involving laparotomy plus right thoracotomy with intrathoracic anastomosis. The stomach was typically fashioned into a gastric conduit approximately 3 to 4 cm in width. Reconstruction routes were categorized as either the posterior mediastinal path or alternative non–esophageal-bed routes including the retrosternal or subcutaneous positions. Reflux symptoms were measured using QLQ-OES18 items 14 and 15 on a 1–4 scale with a one-week recall period [13]. Symptom-positive reflux was defined as ≥ 2 (“a little” or more) on either item, consistent with the validated cutoff proposed by Lagergren et al [14]. Time-to-event analyses used the first occurrence within 12 months, and longitudinal severity was modeled as an ordered outcome over time.

### Handling of missing data

Missingness was evaluated using Little’s MCAR test; assuming patterns were compatible with MAR, we performed multiple imputation by chained equations (MICE). Imputation was restricted to visits within each patient’s observed follow-up window; observations occurring after the last attended follow-up visit were not imputed. The imputation model included all variables used in the Cox and mixed-effects analyses, and estimates were combined using Rubin’s rules.

### Predictor selection and model specification

Before model development, a directed acyclic graph reflecting the theoretical relationships between candidate variables and postoperative reflux was prespecified based on prior literature, clinical knowledge, and the temporal ordering of variables, and analyzed using DAGitty [15]. We prespecified 10 clinically important candidate predictors. Under the DAGitty “Adjustment (total effect)” framework, each predictor was treated in turn as the exposure and postoperative reflux as the outcome to identify exposure-specific minimal sufficient adjustment sets. Arrow directions were prespecified based on prior clinical knowledge, causal assumptions, and the temporal ordering of variables. Variables appearing in these adjustment sets were used to identify key potential confounding structures and to refine the initial candidate predictor pool.

For the longitudinal severity analysis, an ordinal generalized linear mixed model (GLMM) with a cumulative logit link function was specified. This approach was selected to explicitly model the ordinal nature of the symptom scores while accounting for the non-independence of repeated measurements derived from the same patient. GLMM predictors were chosen from prespecified clinically relevant candidate variables, guided by DAG-derived confounding structures and preliminary univariable screening (P ≤ 0.10) [16]. Additionally, time-by-covariate interactions were tested to evaluate how the impact of predictors on symptom severity evolved over the follow-up period.

For the time-to-event analysis, stability selection LASSO was applied to prespecified clinical predictors and DAG-informed covariates, retaining variables with a selection frequency ≥ 0.70. Selected predictors were entered into the Cox model. Proportional hazards were assessed using Schoenfeld residuals, and nonproportional covariates were modeled with time-varying coefficients using interactions with log(time).

To facilitate bedside use, we implemented the final time-varying Cox model as a web-based calculator that outputs individualized cumulative reflux risk at prespecified postoperative time points and assigns patients to low/intermediate/ high-risk groups to guide the timing and intensity of symptom surveillance.

### Survival curves, model performance and longitudinal analysis

Median follow-up was estimated by reverse Kaplan–Meier analysis, and Kaplan–Meier curves were used to estimate the cumulative incidence of first symptom-positive reflux at 1, 3, 6, and 12 months after surgery. Model performance was evaluated in the development and temporal external validation cohorts. Discrimination was assessed by time-dependent ROC analysis using the model linear predictor to calculate AUCs at 1, 3, and 6 months. Calibration was evaluated by calibration curves, and decision curve analysis was used to assess net clinical benefit against treat-all and treat-none strategies. In the longitudinal ordinal GLMM, between-patient heterogeneity was summarized by the intraclass correlation coefficient (ICC).

### Statistical methods

Descriptive statistics were summarized according to data distribution. Normally distributed continuous variables were expressed as mean ± standard deviation and compared using Student’s t-test, while non-normally distributed variables were presented as median with interquartile range (IQR) and compared using the Wilcoxon rank-sum test. Categorical variables were summarized as frequencies and percentages, with group comparisons performed using the chi-square test or Fisher’s exact test as appropriate. All statistical tests were two-sided, with significance set at P < 0.05. All analyses were performed in R 4.5.1, mainly using the survival, timeROC, rms, mice, glmnet, and glmmTMBpackages.

## Results

### Patient characteristics and reflux outcomes

**Fig. 1:**
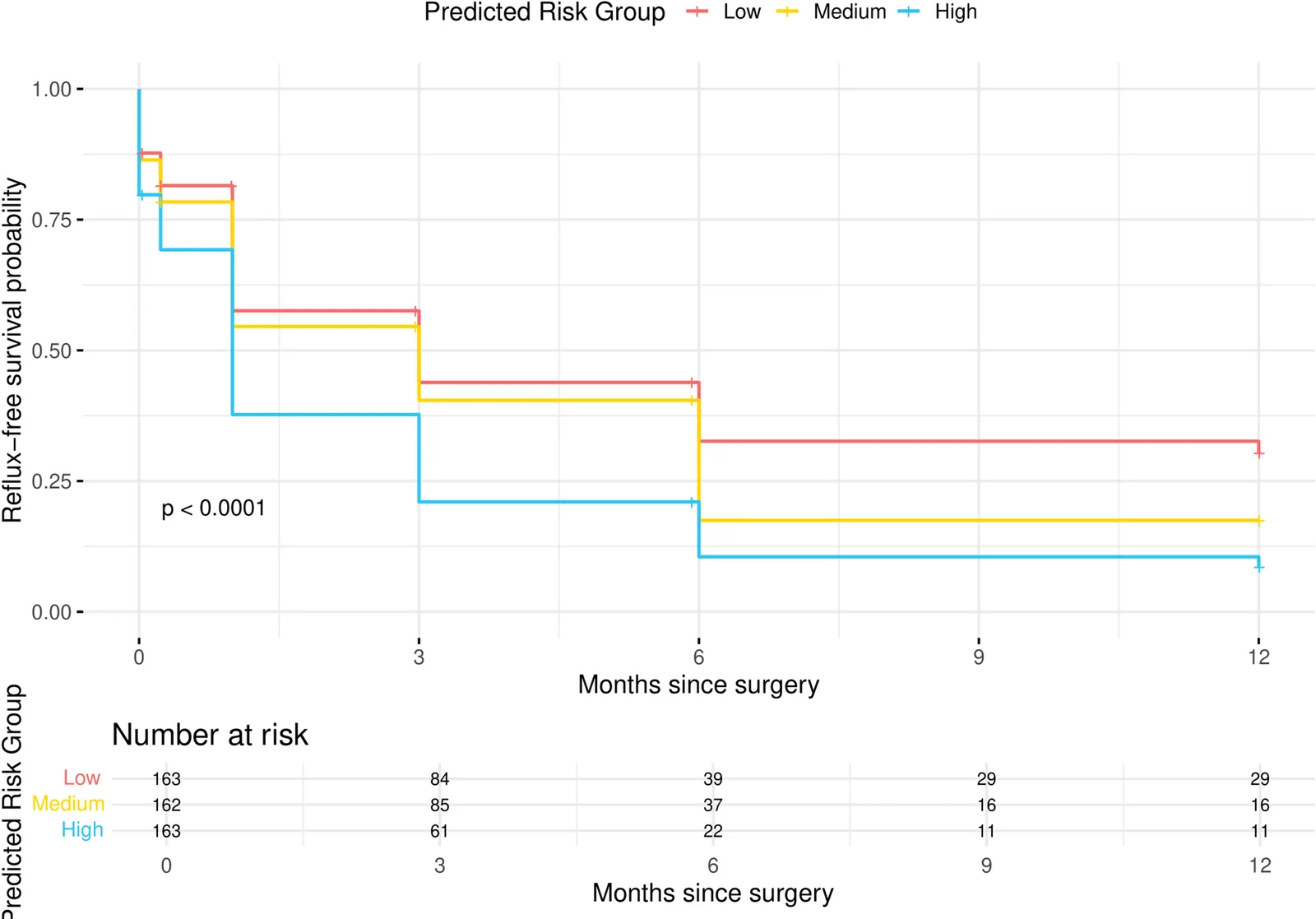
Reflux-free survival by model-predicted risk group. Kaplan–Meier curves stratified by predicted risk (Low = red, Medium = yellow, High = blue). Log-rank p < 0.0001. Numbers at risk are shown at 1, 3, 6, 9, and 12 months; higher predicted risk corresponds to earlier reflux events and lower reflux-free survival

A total of 488 of 527 screened patients were included in the derivation cohort (Supplementary Fig. 1), and 143 patients were included in the temporal external validation cohort. Baseline demographic and clinicopathologic characteristics of the two cohorts are summarized in Table 1. Missing data were handled using multiple imputation, and the observed and imputed distributions showed acceptable consistency (Supplementary Fig. 2). Median follow-up was 12 months. The median time to first clinically significant reflux was 6 months (IQR 1–12), and the cumulative incidence of first clinically significant reflux was 50.6% (95% CI 45.9–54.8) at 1 month, 66.7% (62.2–70.6) at 3 months, 81.8% (77.4–85.4) at 6 months, and 82.8% (78.4–86.4) at 12 months. Figure 1 shows the reflux-free survival curves stratified by risk tertiles derived from the Cox prognostic index.

**Table 1:** Baseline demographics and clinicopathologic characteristics of the derivation and external validation cohorts

Variables	Total	Derivation (n = 488)	Validation (n = 143)	P-value
Age (median [IQR])	63.77 (56.69–69.00)	63.67 (56.00–68.99)	63.78 (58.64–70.58)	0.079
BMI (median [IQR])	22.86 (20.72–25.15)	22.77 (20.70–24.79)	23.23 (20.96–25.45)	0.130
Oral-to-intake (median [IQR])	9.00 (2.00–21.00)	9.00 (4.00–19.00)	17.00 (2.00–21.00)	0.367
Pack years (median [IQR])	15.00 (0.00–30.00)	15.00 (0.00–30.00)	15.00 (0.00–30.00)	0.662
Alcohol dose (median [IQR])	1500.00 (0.00–5600.00)	1500.00 (0.00–6000.00)	1500.00 (0.00–5000.00)	0.700
Weight Change Pct (median [IQR])	0.00 (-0.03–0.02)	0.00 (-0.03–0.02)	0.00 (-0.02–0.02)	0.009
PGSGA_admit (median [IQR])	3.00 (2.00–6.00)	3.00 (2.00–6.00)	3.50 (2.00–5.75)	0.478
PGSGA_preop (median [IQR])	3.00 (2.00–6.00)	3.00 (2.00–6.00)	4.00 (2.75–5.75)	0.392
PGSGA_discharge (median [IQR])	6.00 (5.00–7.00)	6.00 (5.00–7.00)	4.50 (4.00–6.00)	0.092
Operation duration (median [IQR])	230.00 (205.00–263.50)	230.00 (205.00–250.00)	245.00 (191.25–305.00)	0.002
Blood loss (median [IQR])	100.00 (100.00–200.00)	100.00 (100.00–200.00)	200.00 (100.00–200.00)	< 0.001
Gender				0.370
Female	115(18.23%)	93 (19.06%)	22 (15.38%)	
Male	514 (81.46%)	393 (80.53%)	121 (84.62%)	
Missing	2 (0.32%)	2 (0.41%)	0 (0.00%)	
ECOG				0.013
0	529 (83.84%)	399 (81.76%)	130 (90.91%)	
1	102 (16.16%)	89 (18.24%)	13 (9.09%)	
Neoadjuvant				< 0.001
No	361 (57.21%)	311 (63.73%)	50 (34.97%)	
Yes	270 (42.79%)	177 (36.27%)	93 (65.03%)	
Hypertension				0.187
No	540 (85.58%)	423 (86.68%)	117 (81.82%)	
Yes	91 (14.42%)	65 (13.32%)	26 (18.18%)	
Diabetes				0.384
No	573 (90.81%)	440 (90.16%)	133 (93.01%)	
Yes	58 (9.19%)	48 (9.84%)	10 (6.99%)	
Surgical approach				1.000
McKeown	574 (90.97%)	443 (90.78%)	131 (91.61%)	
Ivor Lowis	54 (8.56%)	42 (8.61%)	12 (8.39%)	
Missing	3 (0.48%)	3 (0.61%)	0 (0.00%)	
Anastomosis type				0.576
circle	511 (80.98%)	398 (81.56%)	113 (79.02%)	
S–S	120 (19.02%)	90 (18.44%)	30 (20.98%)	
Tumor location				< 0.001
upper	52 (8.24%)	37 (7.58%)	15 (10.49%)	
middle	313 (49.60%)	264 (54.10%)	49 (34.27%)	
lower	229 (36.29%)	165 (33.81%)	64 (44.76%)	
EGJ	37 (5.86%)	22 (4.51%)	15 (10.49%)	
Recon organ				0.039
Stomach	605 (95.88%)	465 (95.29%)	140 (97.90%)	
jejunum	25 (3.96%)	23 (4.71%)	2 (1.40%)	
transverse colon	1 (0.16%)	0 (0.00%)	1 (0.70%)	
Recon route				0.038
posterior mediastinal route	609 (96.51%)	467 (95.70%)	142 (99.30%)	
retrosternal route	22 (3.49%)	21 (4.30%)	1 (0.70%)	
pTNM				< 0.001
Stage 0	111 (25.17%)	111 (27.75%)	0 (0.00%)	
Stage I	71 (16.10%)	62 (15.50%)	9 (21.95%)	
Stage II	131 (29.71%)	114 (28.50%)	17 (41.46%)	
Stage III	110 (24.94%)	95 (23.75%)	15 (36.59%)	
Stage IV	18 (4.08%)	18 (4.50%)	0 (0.00%)	
Missing	190 (30.11%)	88 (18.03%)	102 (71.33%)	
Histology				0.797
SCC	604 (96.64%)	466 (96.48%)	138 (97.18%)	
AC	21 (3.36%)	17 (3.52%)	4 (2.82%)	
Missing	6 (0.95%)	5 (1.02%)	1 (0.70%)	
Anastomotic leak				0.231
No	568 (90.02%)	435 (89.14%)	133 (93.01%)	
Yes	63 (9.98%)	53 (10.86%)	10 (6.99%)	
Hoarseness				< 0.001
No	603 (95.56%)	460 (94.26%)	143 (100.00%)	
Yes	28 (4.44%)	28 (5.74%)	0 (0.00%)	

^*^ Weight Change Pct represents the percentage of body weight change from admission to discharge

^*^ Pack years calculated as: years of smoking × (cigarettes per day ÷ 20). Alcohol dose (g/years) calculated as: daily alcohol intake (g/day) × years of drinking

^*^ Blood loss refers to the volume of intraoperative bleeding

^*^ PGSGA (Patient-Generated Subjective Global Assessment) is a nutrition assessment tool; higher scores indicate greater risk of malnutrition

^*^ S–S = side-to-side anastomosis; circ. = circular stapled anastomosis;

^*^ *AC*: Adenocarcinoma; *SCC*: Squamous-cell carcinoma;

^*^ pTNM staging based on the latest AJCC/UICC esophageal cancer system (8th edition) [39]

### DAG-derived adjustment sets and candidate pool

Using DAGitty, exposure-specific minimal sufficient adjustment sets were derived for the 10 core candidate predictors. No additional adjustment was required when age, sex, or neoadjuvant therapy was treated as the exposure, whereas the remaining predictors required adjustment for DAG-identified confounders. The DAG structures are shown in Supplementary Fig. 3, and the detailed adjustment sets are provided in Supplementary Table 1. Variables appearing in one or more adjustment sets were further summarized in Supplementary Table 2 to clarify potential confounding structures and refine the candidate predictor pool.

### Predictors of early clinically significant reflux

For the time-to-event analysis, predictor selection was guided by a prespecified directed acyclic graph to identify the minimal sufficient adjustment set and a stability-selection LASSO procedure. To prevent overfitting, the LASSO method shrinks coefficients toward zero as the penalty parameter λ increases, effectively retaining only the most robust predictors. This process identified six key variables: BMI, neoadjuvant therapy, tumor location, oral-to-intake interval, age, and alcohol dose (Supplementary Table 3; Supplementary Fig. 4). Predictors retained by LASSO from the DAG-informed candidate pool were entered into the multivariable Cox model. The proportional hazards assumption was violated for both age and BMI (global p < 0.001; Supplementary Table 4, and these variables were therefore modeled with time-varying coefficients in the final model. To further illustrate their time-dependent effects, we plotted the postoperative log-HR curves with 95% confidence intervals for age and BMI (Supplementary Fig. 5). Older age was associated with a lower reflux risk, and this protective effect became stronger over time, whereas higher BMI was associated with a higher reflux risk, and this adverse effect increased over time.

In the final multivariable model, distal/EGJ tumor location (HR = 1.931, 95%CI 1.562 ~ 2.387) and a higher BMI (HR = 1.312, 95% CI 1.181 ~ 1.458) were independently associated with an increased hazard of early reflux. Conversely, neoadjuvant therapy (HR = 0.549, 95% CI 0.438 ~ 0.687), older age (HR = 0.842, 95% CI 0.762 ~ 0.930), and a longer oral-to-intake (HR = 0.821, 95% CI 0.742 ~ 0.909) were significantly protective. Alcohol dose was not a significant predictor (Table 2).

**Table 2:** Multivariable ordinal mixed-effects model for postoperative reflux symptom severity

Variable	β	SE	OR	95% CI	P value
Follow-up month (spline terms)					
ns(FollowupMonth, 3)1	1.097	0.186	2.995	2.079–4.315	< 0.001
ns(FollowupMonth, 3)2	2.623	0.229	13.778	8.802–21.566	< 0.001
ns(FollowupMonth, 3)3	-0.337	0.161	0.714	0.521–0.978	0.036
Main effects					
Age	0.108	0.097	1.114	0.922–1.346	0.262
BMI	0.084	0.092	1.088	0.908–1.302	0.360
Oral to intake	-0.115	0.098	0.891	0.735–1.080	0.241
Pack-years	-0.125	0.095	0.882	0.732–1.064	0.189
Gender					
Male	reference			–	–
Female	0.291	0.110	1.337	1.077–1.660	0.008
Tumor location					
Proximal (upper / middle)	reference			–	–
Distal (lower / EGJ)	0.620	0.090	1.859	1.559–2.218	< 0.001
Anastomosis type					
circ	reference		1.000	–	–
S–S	0.153	0.119	1.165	0.923–1.470	0.198
Neoadjuvant therapy					
No	reference		1.000	–	–
Yes	-0.644	0.098	0.525	0.433–0.637	< 0.001
Time-varying effects					
ns(FollowupMonth, 3)1 × Age	-0.186	0.180	0.830	0.583–1.183	0.303
ns(FollowupMonth, 3)2 × Age	-0.971	0.228	0.379	0.242–0.592	< 0.001
ns(FollowupMonth, 3)3 × Age	0.012	0.157	1.012	0.743–1.377	0.942
ns(FollowupMonth, 3)1 × BMI	0.269	0.184	1.309	0.913–1.877	0.143
ns(FollowupMonth, 3)2 × BMI	0.709	0.220	2.032	1.321–3.125	0.001
ns(FollowupMonth, 3)3 × BMI	0.031	0.151	1.031	0.767–1.387	0.839
ns(FollowupMonth, 3)1 × Oral to intake	0.410	0.189	1.507	1.040–2.183	0.030
ns(FollowupMonth, 3)2 × Oral to intake	-0.344	0.229	0.709	0.453–1.110	0.133
ns(FollowupMonth, 3)3 × Oral to intake	0.123	0.157	1.131	0.831–1.540	0.434
ns(FollowupMonth, 3)1 × Pack-years	0.029	0.181	1.030	0.722–1.468	0.871
ns(FollowupMonth, 3)2 × Pack-years	0.109	0.224	1.115	0.719–1.729	0.627
ns(FollowupMonth, 3)3 × Pack-years	0.072	0.153	1.075	0.796–1.451	0.639

### Prediction model performance

**Fig. 2:**
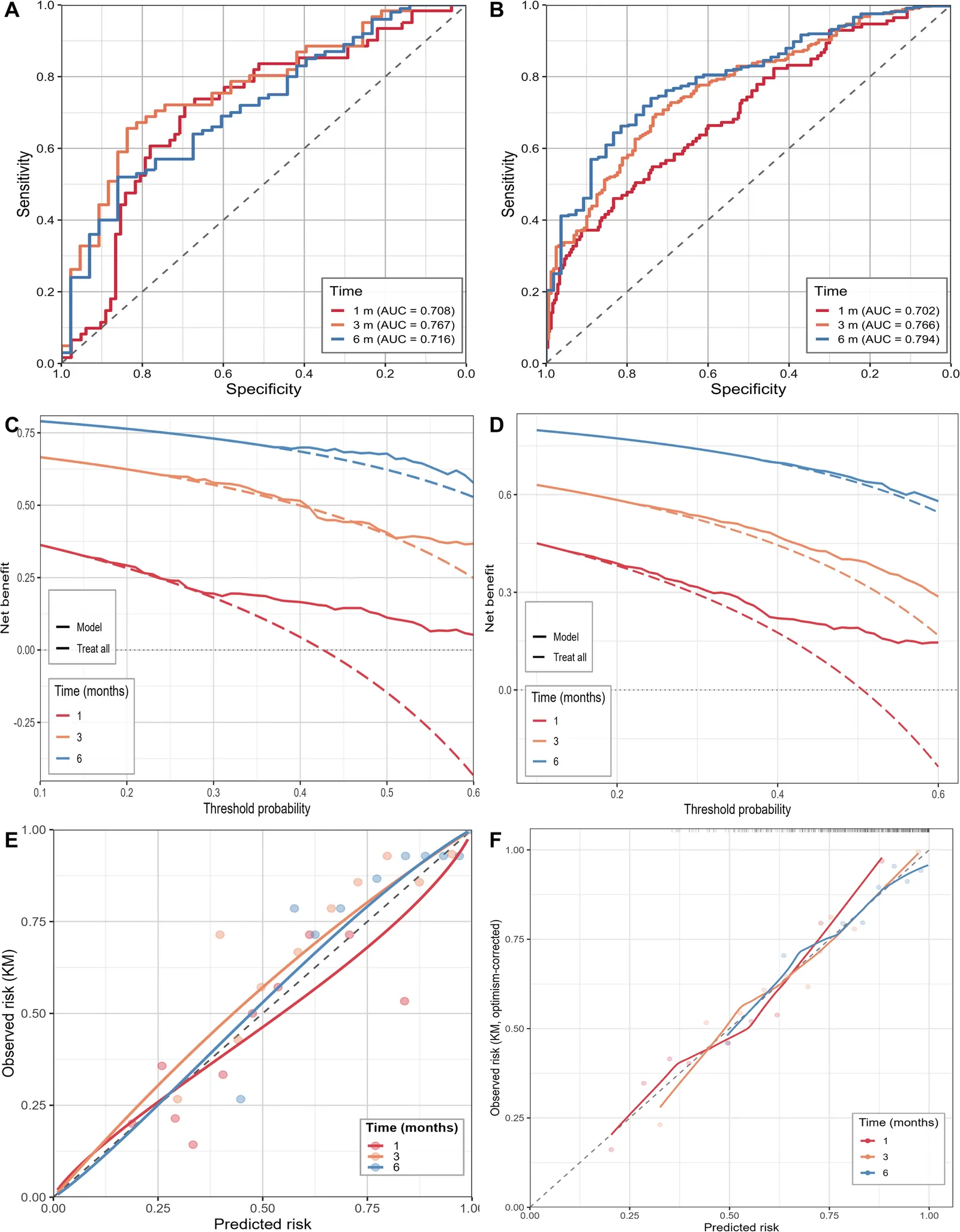
Model performance for predicting post-esophagectomy reflux: Internal derivation and external validation cohort results. Left column (ACE) = external validation; right column (BDF) = internal derivation. A–B: ROC at 1, 3, 6 months. C–D: Decision curve analysis (DCA). E–F: Calibration (observed vs predicted)

The time-varying Cox model showed good discrimination. In the development cohort, the time-dependent AUCs at 1, 3, and 6 months were 0.702 (95% CI 0.644–0.761), 0.766 (95% CI 0.720–0.811), and 0.794 (95% CI 0.735–0.852), respectively; the corresponding values in the external validation cohort were 0.708 (95% CI 0.621–0.796), 0.767 (95% CI 0.676–0.859), and 0.716 (95% CI 0.627–0.804) (Supplementary Table 5). Calibration curves showed good agreement between predicted and observed risks at 1, 3, and 6 months in both cohorts, with overall performance close to the ideal reference line. Decision curve analysis further showed net clinical benefit over a wide range of threshold probabilities at 1, 3, and 6 months, compared with the treat-all and treat-none strategies **(**Fig. 2**)**.

### Clinical implementation: a web-based risk calculator

**Fig. 3:**
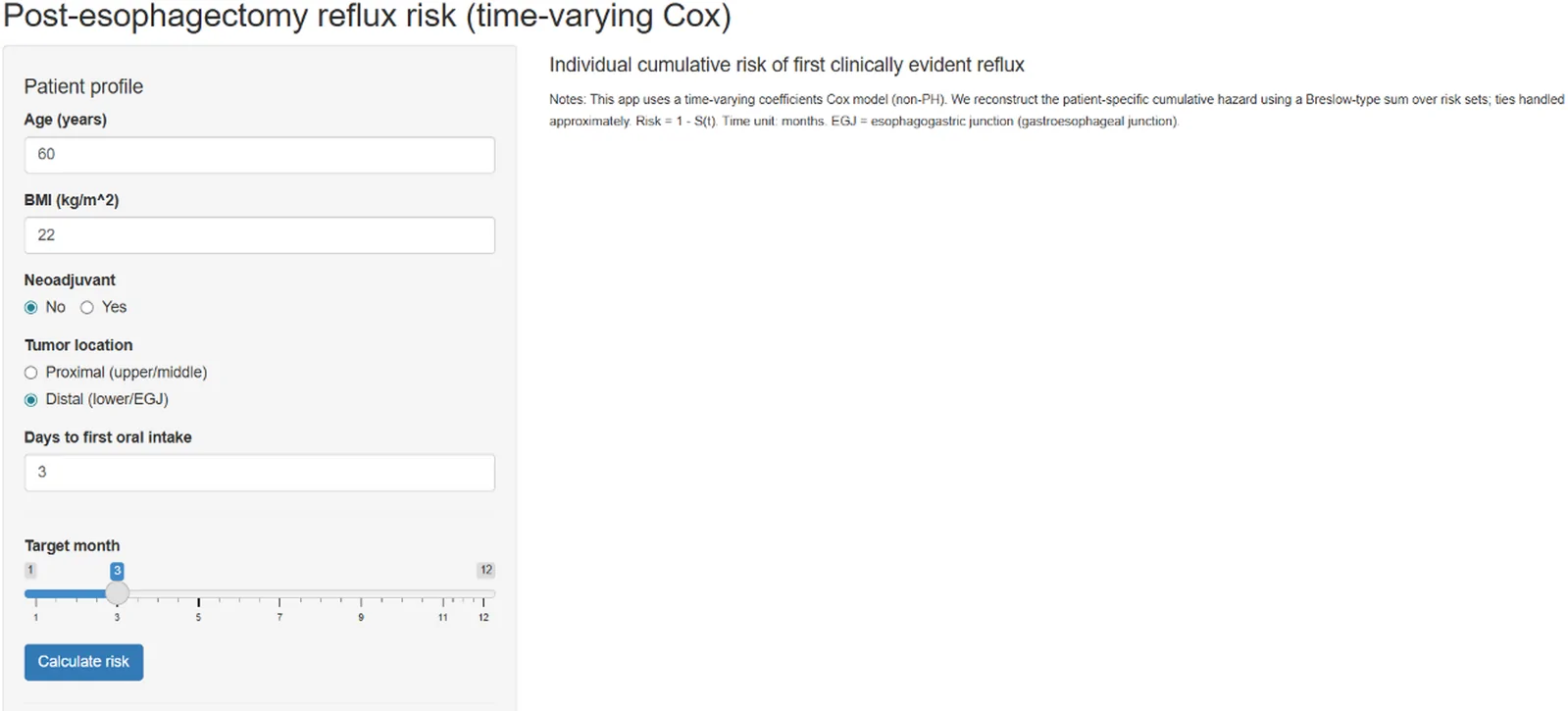
Dynamic early reflux risk calculator after esophagectomy (time-varying Cox model)

To facilitate clinical application and bedside use, the dynamic Cox model was deployed as a publicly accessible, interactive web-based calculator. The tool uses five readily available predictors (age, BMI, tumor location, neoadjuvant therapy, and oral-to-intake) to generate an individualized, time-sensitive risk score. Due to the model’s time-varying coefficients, which preclude a static nomogram, the calculator directly outputs the predicted cumulative risk (%) of reflux onset at user-specified postoperative time points **(**Fig. 3**)**. The tool is available at: https://44l2x2-0-0.shinyapps.io/reflux_nomogram_tvc_clean/.

### Longitudinal reflux symptom trajectories and associated factors

Guided by univariable screening (p < 0.10; Supplementary Table 6) and a prespecified DAG, key predictors were included in the final GLMM. The model confirmed a non-linear trend where symptom burden peaked early postoperatively before attenuating. Independent of time, neoadjuvant therapy was protective (OR 0.525, 95% CI 0.433–0.637), whereas distal/EGJ tumors (OR 1.859, 1.559–2.218, p < 0.001) and female sex (OR 1.337, 1.077–1.660) were associated with greater severity. Although age, BMI, and oral-to-intake were not significant as main effects, significant time–covariate interactions revealed that early symptom escalation was slower in older patients (interaction OR 0.379) but steeper in those with higher BMI (OR 2.032, p = 0.001) or a shorter oral-to-intake time (OR 1.507, p = 0.030). Full results are detailed in Table 3**.**

**Table 3:** Multivariable cox proportional-hazards model for factors associated with time to first post-operative reflux episode

Variable	β	SE	HR (95% CI)	P value
BMI	0.272	0.054	1.312 (1.181–1.458)	< 0.001
Age	-0.173	0.051	0.842 (0.762–0.930)	0.0007
Tumor location				
Proximal (upper / middle)	Reference	—	—	—
Distal (lower / EGJ)	0.658	0.108	1.931 (1.562–2.387)	< 0.001
Neoadjuvant therapy				
No	Reference	—	—	—
Yes	-0.600	0.115	0.549 (0.438–0.687)	< 0.001
Oral to intake	-0.197	0.052	0.821 (0.742–0.909)	< 0.001
Alcohol dose	-0.000	0.000	1.000 (1.000–1.000)	0.279

The model showed good fit (likelihood-ratio p < 0.001) with a low intraclass correlation coefficient (ICC = 0.039), indicating minimal between-patient clustering.

## Discussion

This study elucidated the temporal trajectory of reflux symptoms following esophagectomy, revealing that symptoms were most pronounced 1–3 months postoperatively before declining and plateauing. In our cohort, the cumulative incidence of clinically significant reflux reached 50.6% within the first postoperative month. This finding aligns closely with the 59.9% incidence reported by Liu et al [17]. during oral feeding rehabilitation. This consistency is attributable to our shared use of the QLQ-OES18 scale and a specific focus on capturing all symptomatic events rather than solely severe pathologies, an approach that accurately reflects the ubiquity of early postoperative reflux. This trend likely reflects restored gastric motility, resolved inflammation, and patients’ adoption of compensatory strategies. Nevertheless, reflux remains a persistent burden on quality of life, compromising intake and sleep [18]. Furthermore, given the potential for cumulative complications like esophagitis and Barrett’s metaplasia [19], reflux warrants continuous postoperative management. To address this need, we not only identified key factors associated with early reflux onset and longitudinal severity but also integrated them into a validated, web-based prediction tool. By translating complex risk profiles into a user-friendly calculator, we aim to assist nurses in shifting from reactive symptom management to proactive, personalized surveillance. To our knowledge, this is the first reflux risk prediction model to undergo both internal and external validation.

By integrating both time-to-event and longitudinal analyses, we revealed distinct risk profiles for early reflux onset versus sustained symptom severity. Anatomical factors played a dominant role in early risk. Distal/EGJ tumors significantly increased reflux hazard, likely due to the extensive disruption of the anti-reflux barrier and lower anastomosis level [20, 21]. Higher BMI was also associated with an elevated risk of early reflux events, which aligns with previous research [22]. Further time-varying coefficient analysis suggested that the effect of BMI on postoperative reflux was not constant but became stronger over time. Together with the multivariable Cox results, this indicates that higher BMI was associated with increased reflux risk and that its adverse effect may persist during postoperative recovery. This finding is consistent with previous studies and may be partly explained by obesity-related increases in intragastric pressure, gastroesophageal pressure gradient, and impairment of the anti-reflux barrier [23, 24]. Clinically, patients with higher BMI may require more sustained monitoring and management during recovery. Neoadjuvant therapy emerged as a protective predictor of early reflux. One possible biological explanation is that tumor regression after multimodal therapy may reduce the extent of dissection at the hiatus and may preserve some anti reflux anatomy [25]. Hasan et al [22]. reported that the absence of perioperative chemoradiation was associated with worse patient reported reflux after esophagectomy, which is directionally consistent with our findings. However, this association requires caution because residual confounding and patient selection may still contribute in an observational study [26]. The study used a prespecified DAG to adjust for key confounders. The study also evaluated transportability by temporal validation. Neoadjuvant therapy use differed between the derivation cohort and the validation cohort, and the model performance remained stable.

GLMM analysis suggested that age significantly shaped postoperative reflux symptom trajectories. Younger patients reported symptoms earlier and more prominently, whereas older patients had a lower symptom burden in the early postoperative period. This pattern was directionally consistent with the time-varying Cox model. Previous studies have shown that increasing age may be associated with reduced perception of GERD symptoms and decreased esophageal sensitivity to acid, which may partly explain the milder early symptoms observed in older patients [27, 28]. However, this should not be interpreted as implying a lower need for long-term management in older adults. Dysphagia in older adults is associated with increased risks of aspiration, pneumonia, and malnutrition [29, 30], and persistent nutritional decline is common after esophagectomy [31]. Therefore, once reflux-related complications occur, older patients may be more vulnerable to adverse recovery outcomes. While sex did not predict early reflux onset, the GLMM identified female sex as a risk factor for greater longitudinal severity. This disparity may stem from anatomical differences, such as a shorter post-reconstruction esophagus, or increased visceral pain sensitivity associated with post-menopausal hormonal changes [22, 32]. This disparity could stem from hormonal influences or differences in pain processing pathways. Our analysis also clarified the complex relationship between early oral feeding and symptom burden. Specifically, the Cox model linked shorter time to first oral intake with a higher hazard of early reflux, while the GLMM confirmed that earlier feeding correlated with greater initial symptom intensity. However, these findings do not diminish the value of enhanced recovery protocols [33]. Instead, early feeding likely acts as a physiological challenge, exposing susceptible patients to dietary triggers before gut adaptation occurs [5]. Consistent with previous observations that recumbency after eating exacerbates symptoms [9], this transient surge in patient-reported intensity underscores the need for proactive management. Consequently, reflux-prevention strategies should be initiated concurrently with the start of oral intake rather than waiting for symptoms to escalate.

From a strategic perspective, our study employed a comprehensive assessment framework to maximize clinical utility. Regarding the reflux definition, we utilized a sensitive cutoff of score 2 or higher on the QLQ-OES18 scale. This choice aligns with a proactive screening strategy where capturing even mild symptoms facilitates early behavioral interventions such as dietary adjustments before complications arise. Although patient-reported outcomes may not perfectly correlate with objective pH monitoring [34], they directly reflect the symptom burden impacting daily life. Furthermore, to capture recovery dynamics beyond binary outcomes, we integrated a generalized linear mixed model to map the longitudinal trajectory of symptom severity [35]. While the time-varying Cox model predicts the timing of symptom onset, the mixed model characterizes the evolution of symptom intensity. This distinction is clinically critical as it differentiates transient mild episodes from persistent severe burdens requiring long-term intervention. Consequently, our web-based calculator synthesizes these probability and severity dimensions into a holistic tool for personalized surveillance.

While symptom management is intensive during hospitalization, the transition to outpatient care is often unstructured, leaving many survivors with unmet needs [36]. Furthermore, uncertainty regarding the optimal timing and intensity of monitoring hinders standardized follow-up and efficient resource allocation [11]. In this context, our risk prediction model helps direct follow-up resources to patients most likely to experience burdensome reflux. High-risk profiles include distal tumor location, elevated BMI and earlier resumption of oral feeding, and age extremes also warrant attention: younger patients often have more pronounced early symptoms, whereas older patients may have under-recognized reflux. These groups should receive more intensive, nurse-led surveillance and support after discharge, with individualized reflux-prevention plans initiated before discharge and clear explanations of precautionary measures, alarm symptoms and when to contact the care team [37]. Patients with lower predicted risk can be followed at a reduced frequency with core safety checks maintained, minimizing unnecessary clinic visits and improving use of follow-up capacity. Specifically, nursing teams can utilize this tool prior to discharge to inform patients about their individual risks and to guide personalized behavioral interventions, such as tailored dietary modifications and weight management strategies for high-risk patients [38]. To ensure accessibility, the web-based calculator is publicly available and features a simplified interface that requires no specialized statistical knowledge. Clinicians simply input five routine variables to generate cumulative risk estimates, facilitating rapid bedside decision-making. In conclusion, this web-based calculator is specifically designed for bedside application by nursing teams before patient discharge. It can stratify patients into different risk categories according to their individualized probability of developing early postoperative reflux, and thus shifts the management of reflux after esophagectomy from reactive, symptom-triggered intervention to proactive, risk-guided care and support.

This study has limitations. First, as a single-center analysis of predominantly squamous cell carcinoma patients undergoing McKeown esophagectomy, generalizability to Western populations with adenocarcinoma or Ivor Lewis procedures requires validation. However, the consistency of our core predictors with Western data suggests cross-population applicability [22]. Second, our reflux definition relies on patient-reported symptoms (score ≥ 2) rather than objective pH monitoring. While this validated threshold captures subjective burden for screening, it may not perfectly correlate with physiological acid exposure. Third, unstandardized perioperative pyloric management prevented evaluation of its impact. Finally, although the proportional odds assumption for the mixed-effects model was not strictly met for all predictors, the low ICC (0.039) and consistent directionality of effects support the model’s clinical interpretability.

## Conclusion

We integrated time to first clinically significant reflux with longitudinal symptom severity and developed a time-sensitive risk assessment tool and web-based calculator using routinely available pre-discharge variables, with temporal validation. Distal or esophagogastric junction tumors, higher BMI, earlier resumption of oral intake, and younger age identified survivors at higher risk of early or persistent reflux, while women showed a tendency toward worsening symptoms over time and neoadjuvant therapy was associated with lower risk. Embedding this tool into survivorship care pathways could enable targeted early symptom screening, timely patient education and supportive care, and escalation of interventions when needed. The ultimate goal is to reduce reflux-related illness burden and preserve long-term health-related quality of life after esophagectomy.

## Supplementary Information

Below is the link to the electronic supplementary material.

Supplementary file1 (DOCX 27 KB)

Supplementary file2 (DOCX 3850 KB)
