# Supplementary material for: Clinical predictors of early reflux and symptom severity after esophagectomy: a risk prediction tool
Source: Esophagus. 2026 Jun 30;23(4):963–74. doi: 10.1007/s10388-026-01225-z (PMC13723491; doi:10.1007/s10388-026-01225-z)
Supplement: Supplementary file 1 [file 10388_2026_1225_MOESM1_ESM.docx]

**TABLE**

**Supplementary Table 1. Exposure-specific minimal sufficient adjustment sets derived from the prespecified DAG using DAGitty**

| Prespecified candidate predictor treated as exposure | Outcome | Minimal sufficient adjustment set(s) identified in DAGitty | Adjustment required |
| --- | --- | --- | --- |
| Age | Reflux | None | No |
| Gender | Reflux | None | No |
| Pathology | Reflux | Age | Yes |
| FeedingStartTime | Reflux | AnastomoticLeak, ECOG | Yes |
| SurgicalApproach | Reflux | Age, Neoadjuvant, TumorLocation; Age, TumorLocation, pTNM; Pathology, TumorLocation, pTNM | Yes |
| WeightChangePct | Reflux | AnastomoticLeak, PGSGA_Admission, PGSGA_Discharge | Yes |
| AnastomosisType | Reflux | Age, Neoadjuvant, SurgicalApproach; Age, SurgicalApproach, pTNM; SurgicalApproach, TumorLocation | Yes |
| Neoadjuvant | Reflux | None | No |
| TumorLocation | Reflux | Age | Yes |
| BMI | Reflux | Age | Yes |

**Supplementary Table 2. Variables appearing in one or more exposure-specific minimal sufficient adjustment sets and their role in model development**

| Variable | Appeared in adjustment set(s) for which exposure(s) | Role in model development |
| --- | --- | --- |
| Age | Pathology, SurgicalApproach, AnastomosisType, TumorLocation, BMI | Used to inform candidate pool and confounding structure |
| AnastomoticLeak | FeedingStartTime, WeightChangePct | Used to inform candidate pool and confounding structure |
| ECOG | FeedingStartTime | Used to inform candidate pool and confounding structure |
| Neoadjuvant | SurgicalApproach, AnastomosisType | Used to inform candidate pool and confounding structure |
| TumorLocation | SurgicalApproach, AnastomosisType | Used to inform candidate pool and confounding structure |
| pTNM | SurgicalApproach, AnastomosisType | Used to inform candidate pool and confounding structure |
| Pathology | SurgicalApproach | Used to inform candidate pool and confounding structure |
| PGSGA_Admission | WeightChangePct | Used to inform candidate pool and confounding structure |
| PGSGA_Discharge | WeightChangePct | Used to inform candidate pool and confounding structure |
| SurgicalApproach | AnastomosisType | Used to inform candidate pool and confounding structure |

**Supplementary Table 3. Selection Summary of Candidate Variables for Cox Proportional-Hazards Modeling Based on LASSO and DAG Criteria**

| Variable | LASSO Selection Rate | Recommended by DAG |
| --- | --- | --- |
| BMI | 100% | Yes |
| Neoadjuvant | 100% | Yes |
| Tumor location | 100% | Yes |
| Oral to intake | 95% | Yes |
| Age | 92% | Yes |
| Alcohol dose | 70% | No |
| Gender | 40% | Yes |
| pTNM | 40% | Yes |
| Surgery duration | 33% | No |
| Anastomosis type | 33% | Yes |
| Pack years | 28% | No |
| PGSGA_prep | 17% | No |
| Hoarseness | 15% | No |
| PGSGA_discharge | 12% | Yes |
| Weightchange_pct | 11% | Yes |
| Anastomotic leak | 11% | Yes |
| Hypertension | 8% | No |
| ECOG | 7% | Yes |
| Blood loss | 6% | No |
| Diabetes | 6% | No |
| PGSGA_admission | 3% | Yes |

**Supplementary Table 4. Tests of the proportional hazards assumption for the Cox model (Schoenfeld residuals)**

| **Variable** | **chisq** | **P value** |
| --- | --- | --- |
| BMI | 4.412 | 0.036 |
| Age | 23.079 | <0.001 |
| Tumor location | 0.986 | 0.321 |
| Neoadjuvant | 1.944 | 0.163 |
| Oral to Intake | 0.520 | 0.471 |
| GLOBAL | 30.862 | <0.001 |

* P-value < 0.05 indicates that the variable may violate the proportional hazards assumption (i.e., the hazard ratio changes over time).

* The "GLOBAL" row represents the test result of the proportional hazards assumption for the overall model. A P-value < 0.05 here suggests that the model as a whole may have violated the assumption.

**Supplementary Table 5. Time-dependent AUCs with 95% confidence intervals of the time-varying Cox model in the development and external validation cohorts**

| Cohort | Time point (months) | AUC | 95% CI |
| --- | --- | --- | --- |
| internal derivation | 1 | 0.702 | 0.644–0.761 |
|  | 3 | 0.766 | 0.720–0.811 |
|  | 6 | 0.794 | 0.735–0.852 |
| external validation | 1 | 0.708 | 0.621–0.796 |
|  | 3 | 0.767 | 0.676–0.859 |
|  | 6 | 0.716 | 0.627–0.804 |

**Supplementary Table 6. GLMM Univariable Analysis of Factors Associated with Post-operative Reflux Score**

| Variable | OR | CI_lower | CI_upper | P value |
| --- | --- | --- | --- | --- |
| Age | 0.799 | 0.724 | 0.881 | <0.001 |
| Anastomosis type |  |  |  |  |
| circ | Reference | |  |  |
| S-S | 1.432 | 1.129 | 1.817 | 0.003 |
| Anastomotic leak |  |  |  |  |
| No | Reference | |  |  |
| Yes | 0.737 | 0.532 | 1.021 | 0.066 |
| BMI | 1.432 | 1.3 | 1.561 | <0.001 |
| Blood loss | 1 | 0.999 | 1.001 | 0.949 |
| Diabetes |  |  |  |  |
| No | Reference | |  |  |
| Yes | 1.166 | 0.848 | 1.604 | 0.345 |
| ECOG |  |  |  |  |
| 0 | Reference | |  |  |
| 1 | 0.917 | 0.712 | 1.181 | 0.504 |
| Gender |  |  |  |  |
| male | Reference | |  |  |
| female | 1.373 | 1.13 | 1.668 | 0.001 |
| Hoarseness |  |  |  |  |
| No | Reference | |  |  |
| Yes | 1.109 | 0.745 | 1.652 | 0.610 |
| Hypertension |  |  |  |  |
| No | Reference | |  |  |
| Yes | 1.258 | 0.959 | 1.649 | 0.0968 |
| Neoadjuvant |  |  |  |  |
| No | Reference | |  |  |
| Yes | 0.581 | 0.474 | 0.713 | <0.001 |
| Oral to intake | 0.984 | 0.978 | 0.99 | <0.001 |
| PGSGA admission | 0.982 | 0.951 | 1.013 | 0.249 |
| PGSGA discharge | 1.014 | 0.966 | 1.063 | 0.579 |
| PGSGA preop | 0.997 | 0.964 | 1.031 | 0.865 |
| Pack Years | 0.996 | 0.992 | 1 | 0.050 |
| Surgery duration | 1.001 | 0.999 | 1.003 | 0.302 |
| Tumor location |  |  |  |  |
| Distal (lower / EGJ) | Reference | |  |  |
| Proximal (upper / middle) | 0.574 | 0.475 | 0.694 | <0.001 |
| Weight change pct | 3.135 | 0.244 | 40.331 | 0.381 |
| pTNM | 1.024 | 0.941 | 1.114 | 0.587 |
