# Supplementary material for: Clinical predictors of early reflux and symptom severity after esophagectomy: a risk prediction tool
Source: Esophagus. 2026 Jun 30;23(4):963–74. doi: 10.1007/s10388-026-01225-z (PMC13723491; doi:10.1007/s10388-026-01225-z)
Supplement: Supplementary file 2 [file 10388_2026_1225_MOESM2_ESM.docx]

**FIGURE**

**Supplementary Fig 1.** **Study Inclusion Flowchart**

**Supplementary Fig 2. Multiple imputation diagnostics: observed vs imputed covariate distributions**

A. Continuous covariates: observed vs imputed distributions (density overlays)

B. Categorical covariates: observed vs imputed level proportions

**Supplementary Fig 3. Exposure-specific directed acyclic graphs for the 10 core candidate predictors under the prespecified master DAG**

Note: Yellow nodes represent exposures, blue nodes represent outcomes, light green nodes represent ancestors of exposures, light blue nodes represent ancestors of outcomes, pink nodes represent common ancestors of exposures and outcomes, gray nodes represent other variables, and white nodes represent adjustment variables (where applicable). Green lines indicate causal pathways, and pink lines indicate bias pathways.

**Panel A. Age**

**Panel B. Gender**

**Panel. C Pathology**

**Panel. D Feeding-start-time**

**Panel. E Surgical approach**

**Panel. F Weight-change-pct**

**Panel G. Anastomosis type**

**Panel. H Neoadjuvant**

**Panel. I Tumor location**

**Panel. J BMI**

**Supplementary Fig 4. LASSO-Cox Regression for Variable Selection**

A. Coefficient paths of predictors as λ increases. Variables with non-zero coefficients at the optimal λ are selected for the final model.

B. Cross-validation curve showing partial likelihood deviance across log(λ) values. The dashed line marks the optimal λ (lambda.1se), balancing model fit and simplicity.

**Supplementary Fig 5. Time-varying log-hazard ratios for age and BMI in the time-varying Cox model**

Time-varying log-hazard ratios for age and BMI. Solid lines show estimated log-hazard ratios, and shaded areas show 95% confidence intervals.
